# Positive Correlation between Activated CypA/CD147 Signaling and MMP-9 Expression in Mice Inflammatory Periapical Lesion

**DOI:** 10.1155/2019/8528719

**Published:** 2019-03-05

**Authors:** Yan-qing Wang, Jie Zhang, Ling-xin Zhu, Jing-jing Yu, Ming-wen Liu, Shen-ting Zhu, Guo-jing Liu, Bin Peng

**Affiliations:** The State Key Laboratory Breeding Base of Basic Science of Stomatology (Hubei-MOST) and Key Laboratory for Oral Biomedicine Ministry of Education, Wuhan University, Wuhan 430000, China

## Abstract

**Aim:**

Cyclophilin A (CypA)/CD147 signaling plays critical roles in the regulation of inflammation and bone metabolism. This study aimed to investigate the participation of CypA/CD147 in mice periapical lesions progression and its relationship with bone resorption.

**Methodology:**

Periapical lesions were induced by pulp exposure in the first lower molars of 40 C57BL/6J mice. The mice were sacrificed on days 0, 7, 14, 21, 28, 35, 42, and 49. Mandibles were harvested for X-ray imaging, microcomputed tomography scanning, histologic observation, immunohistochemistry, enzyme histochemistry, and double immunofluorescence analysis. Western blot was employed to further detect the related molecular signaling pathways in LPS-stimulated RAW 264.7 cells treated with CypA inhibitor.

**Results:**

The volume and area of the periapical lesions increased from day 0 to day 35 and remained comparably stable until day 49. Immunohistochemistry demonstrated that the CypA expression levels also increased from day 0 to day 35 and decreased until day 49, similar to CD147 expression (*R*^2^ = 0.4423, P < 0.05), osteoclast number (*R*^2^ = 0.5101, P < 0.01), and the expression of osteoclastogenesis-related matrix metalloproteinase 9 (MMP-9) (*R*^2^ = 0.4715, P < 0.05). Serial sections further confirmed the colocalization of CypA and CD147 on osteoclasts with immunohistochemistry. And the distribution of CypA-positive or CD147-positive cells was positively correlated with the dynamics of MMP-9-positive cells by using immunofluorescence analysis. Furthermore, CD147 and MMP-9 expression in RAW 264.7 cells were both downregulated with CypA inhibitor treatment (P < 0.05).

**Conclusions:**

The present study reveals the positive correlation of CypA/CD147 signaling and osteoclast-related MMP-9 expression in mice inflammatory periapical lesions progression. Therefore, intervention of CypA/CD147 signaling could probably provide a potential therapeutic target for attenuating inflammatory bone resorption.

## 1. Introduction

Periapical lesions, which occur as a result of bacterial infection of the dental pulp, are osteolytic bone defects characterized with the host response-mediated inflammation and alveolar bone destruction [1–3]. The local inflammatory reaction is involved in the recruitment of various inflammatory cells and secretion of cytokines, including matrix metalloproteinases (MMPs), IL-1*β*, and IL-6, thereby resulting in osteoclast formation and alveolar bone resorption [4–6]. Osteoclasts are the exclusive cells engaged in cartilage and alveolar bone degrading, thus playing critical roles in pathological bone destruction [7, 8]. However, the specific mechanism of osteoclast-mediated bone resorption in periapical lesion is not fully elucidated.

Cyclophilin A (CypA), as a proinflammatory cytokine secreted by monocytes/macrophages, vascular smooth muscle cells, activated platelets, and endothelial cells, has been well studied in multiple osteolytic diseases recently [9–11]. In response to inflammatory and oxidative stimuli, CypA is released into extracellular tissue spaces, regulates protein folding and intracellular trafficking [12–14], and acts as a signal transducer and activator in skeleton, cartilage, and vasculature [11, 15]. High CypA expression is detected in the serum and synovial fluid of patients with rheumatoid arthritis (RA) and the gingival crevicular fluid of patients with periodontitis; the CypA amount is closely correlated with the severity of disease [10, 16]. The regulatory effect of CypA is activated by binding with its cellular receptor, CD147, which is also referred to as the extracellular MMP inducer [17]. CD147 is a transmembrane glycoprotein belonging to the immunoglobulin superfamily; this glycoprotein plays an important role in inflammation regulation, including neutrophil adhesion, chemotaxis, granule release, and oxidative burst [18, 19]. The CypA/CD147 complex acts as a pivotal proinflammatory signaling pathway in monocytes and promotes MMP-9 production from macrophages [10, 20]. In RA, the direct binding of CypA to CD147 upregulates the adhesive and invasive properties of neutrophils and increases MMP-9 expression [17]. Moreover, CypA blockade counteracts the CypA-dependent MMP-9 secretion in monocytes/macrophages and remarkably reduces the inflammatory cell number, MMP-9 production, and cartilage erosion in mice arthritis model [21]. Osteoclastogenesis-mediated periapical bone resorption is dependent on the induction of inflammatory mediators and the secretion of proteases like MMP-9 [4, 22]. Therefore, we suppose that CypA/CD147 signaling is related with osteoclast formation and bone destruction in inflammatory periapical lesions.

To date, the potential roles of CypA/CD147 signaling in periodontitis, RA, inflammatory cardiomyopathy, and cancer have been studied [17, 20, 23, 24], but no studies have shown whether CypA/CD147 is associated with the pathological process of periapical lesions. In the present study, we aimed to examine the expression of CypA/CD147 signaling pathway and its potential roles in osteoclastogenesis and alveolar bone destruction by using a well-established mouse model of periapical lesions. We assumed that the CypA/CD147 signaling pathway may take part in the development of mice experimental periapical lesions by accelerating MMP-9 expression, osteoclastogenesis, and alveolar bone destruction.

## 2. Materials and Methods

### 2.1. Induction of Experimental Periapical Lesions and Sample Preparation

All animal studies were approved and supervised by the Institutional Animal Care and Use Committee of Wuhan University, China. Forty C57BL/6J mice (male; each weighing approximately 20 g; 5 weeks old) were purchased from the Experimental Animal Center of Hubei Province, China, divided into eight groups randomly, and housed and bred under specific pathogen-free conditions (laboratory) throughout the entire experimental period. All mice were anesthetized by intraperitoneal injections of chloral hydra (0.5 mg/g). The first molar pulps of bilateral mandibular were exposed using a number 1/4 round bur until the bur head sank into the pulp chamber. The exposed pulpal tissues were left open to the oral environment without any protection during the entire experimental period to allow contamination of the root canal system and consequently induce periapical lesion formation. Five mice in each group were sacrificed on day 0 (control), 7, 14, 21, 28, 35, 42, and 49 after pulp exposure. The lower jaws were dissected free of soft tissues, fixed with 4% paraformaldehyde at 4°C for 48 h, and subsequently transferred into 0.5% paraformaldehyde filled in airtight containers for long-term storage. After X-ray imaging and microcomputed tomography (*μ*-CT) scanning, the jaws were rinsed with water for 24 h, decalcified with 10% EDTA for 5–6 weeks at room temperature, dehydrated in graded ethanol solutions, and embedded in paraffin. Seriate sections (thickness, 4 mm) were cut in the mesiodistal direction.

### 2.2. High-Resolution X-Ray Imaging and *μ*-CT Analysis

High-resolution X-ray images photographed by DXS PRO (Bruker Corporation, Billerica, MA, USA) were analyzed with Bruker Molecular Imaging software to evaluate the bone intensity distribution of mice periapical lesions. The black region around the distal root apex indicated relatively low bone intensity. The 2D area of this region was measured using Image-Pro Plus 6.0 (Media Cybernetics, Silver Spring, MD, USA). The lower jaws were scanned by the Scan *μ*-CT 50 imaging system (Scanco Medical, Bassersdorf, Switzerland) to evaluate the periapical bone destruction around the distal root of the first molar. The scanning procedure was operated at 70 kV and 114 mA, with an increment of 34.4 mm, a reconstruction matrix of 1024, and an integration time of 300 ms. 3D image analysis software (VGStudio MAX, Heidelberg, Germany) with an isotropic voxel size of 34.4 mm was used to analyze the scanning data; subsequently, the periapical region volume was determined as described previously [25]. All measurements were performed in a double-blind manner.

### 2.3. Histologic Analysis

Paraffin sections possessed the distal root of the first mandibular molars and exhibited a patent root canal apex representing the central portion of the pulp and root canal were selected for haematoxylin-eosin staining, enzyme histochemistry, immunohistochemistry, and double immunofluorescence labeling. The area of detection included the area of periapical destruction and the periodontal ligament space surrounding the distal root of the first molar. One in every 4 sections was analyzed by light microscopy.

### 2.4. Tartrate-Resistant Acid Phosphatase (TRAP) Assay

To identify osteoclasts, TRAP activity was detected using a TRAP kit (Sigma, St. Louis, MO, USA) as described previously [25]. The sections were rehydrated, rinsed, and incubated in a solution of naphthol AS-BI phosphoric acid and fast garnet GBC for 1 h at 37°C. After incubation, the sections were counterstained with hematoxylin, air dried, and mounted. The sections incubated in substrate-free medium served as controls. TRAP+ cells showing colors ranging from dark red to purple and containing three or more nuclei were identified as osteoclasts.

### 2.5. Immunohistochemistry

According to previous experiments, pepsin (Maixin, Fuzhou, China) was used for 20 min in antigen retrieval. Afterward, the sections were washed and stained using a streptavidin-peroxidase (SP) kit (Maixin, Fuzhou, China) according to the manufacturer's instructions. Briefly, endogenous peroxidase activity was blocked with 3% hydrogen peroxide for 30 min at 37°C. Then sections were incubated with normal serum for 40 min at 37°C and subsequently with rabbit polyclonal antibodies against CypA (1:200, Abcam Biotechnology Inc., Cambridge, UK), rabbit polyclonal antibodies against CD147 (1:200, Abcam Biotechnology Inc., Cambridge, UK), and goat polyclonal antibodies against MMP-9 (1:400, Santa Cruz Biotechnology, Inc., Santa Cruz, CA, USA) overnight at 4°C. The sections were incubated with a biotinylated secondary antibody for 40 min at 37°C and with SP for another 30 min at 37°C. Subsequently, the sections were developed with 3, 30-diaminobenzidine (Zhongshan Biotechnology Co., Beijing, China) and counterstained with hematoxylin. Finally, the sections were observed under a microscope. Sections that were substituted with nonimmune rabbit or goat serum instead of primary antibodies served as negative controls. In each specimen, CypA+, CD147+, and MMP-9+ cells in three randomly selected areas around the root apex (the tissue around the lower one-third part of the roots) were counted under 400× magnification by two independent observers. The average number per field and the average area of the periapical lesions in each group were calculated. Three sections from each jaw were analyzed for cell measurement, and the mean average was calculated from these observations. All measurements were performed in a double-blind manner.

### 2.6. Double Immunofluorescence Labeling

This experiment was performed as previously described [25]. After deparaffinization and rehydration, slices were treated with 0.1% Triton X-100 for 15 min (for CypA only) and incubated with 1% bovine serum albumin for 1 h at 37°C to eliminate nonspecific staining. Without washing, slices were incubated with CypA rabbit polyclonal antibody (1:50) and MMP-9 goat polyclonal antibody (1:100) at 4°C overnight. The sections were washed and incubated with the secondary fluorescein donkey anti-rabbit Dylight 488 (1:200, EarthOx, San Francisco, CA, USA) and donkey anti-goat-CY3 (1:200, Proteintech Group, Wuhan, China) antibodies at 37°C for 1 h. Furthermore, 4′,6-diamidino-2-phenylindole was used to stain the nuclei at 37°C for 5 min. Finally, the sections were washed, incubated with AutoFluo Quencher (Applygen, Beijing, China) for 30 min, and viewed by fluorescent microscopy (Leica, Wetzlar, Germany). A single primary antibody (CypA, CD147 and MMP-9) or PBS was used as the control.

### 2.7. Western Blot Analysis

RAW 264.7 cells were treated with non-LPS and LPS (10 *μ*M, Sigma-Aldrich, China) stimulation [26] in the absence or presence of Cyclosporin A (10 *μ*M, CypA inhibitor, Sigma-Aldrich, China) for 24 h. Cells were washed twice with cold PBS, collected, and centrifuged at 1000 rpm for 5 minutes at 4°C. Total cellular proteins were extracted with RIPA lysis containing protease and phosphatase inhibitor. Proteins were quantified by using Bradford protein assay (Applygen Technologies Inc., Beijing, China). Western blot analysis was processed according to our previous protocols [27]. Specific primary antibodies are anti-MMP-9 and anti-CD147.

### 2.8. Statistical Analysis

All data were subjected to statistical analysis with one-way ANOVA using the SPSS 13.0 (SPSS Inc., Chicago, IL, USA). All metering results are presented as mean values±standard deviation. Correlation analysis was performed on the numbers of CypA+ cells with osteoclasts, MMP-9+ cells, and CD147+ cells. Pearson correlation was used (*a* = 0.05). Statistical significance was considered at P < 0.05.

## 3. Results

### 3.1. Mouse Periapical Lesion Size at Different Stages

**Figure 1:**
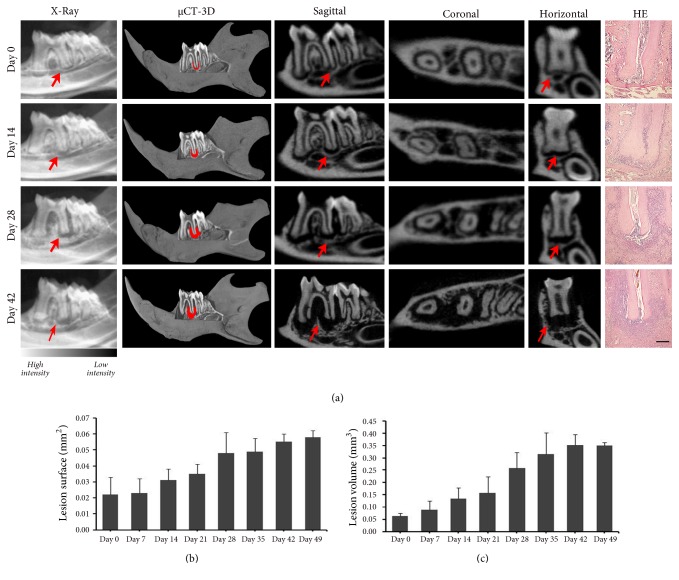
High-resolution x-ray imaging and *μ*CT measurement showed that the range and volume of the periapical region expanded gradually with the time on days 0, 7, 14, 21, 28, 35, 42, and 49. (a) Periapical lesion size was revealed by *μ*CT, high-resolution x-ray imaging and hematoxylin-eosin images. The black area around the root apex means comparably low bone intensity (red arrows). The first molar with the periapical lesions was shown in the sagittal, horizontal, and coronal directions (scale bar = 200 *μ*m). (b) Measurements of periapical lesion volume (mm^3^) in the periapical regions (light blue area). Significant differences were observed at all-time points (P < .05) except on day 42 and 49. (c) Measurements of lesion size (mm^2^). Significant differences were observed at all-time points (P < .05) except between days 0 and 7 and between days 28 and 35.

High-resolution X-ray imaging and *μ*-CT measurement showed that the range and volume of the periapical lesion expanded gradually with time. The periapical lesion around the distal root apex of the mandibular first molar grew in size from day 7 to day 35 and remained stable until day 49 compared with day 0 (Figure 1(a)). The *μ*-CT analysis results showed that the lesions appeared and continuously enlarged in the sagittal, horizontal, and coronal directions from day 0 to day 42 and remained stable until day 49 (Figures 1(b) and 1(c)). The lesion volume was significantly different between day 21 and day 35, day 42, and day 49 (P < 0.05). No statistically significant differences were detected among continuous time points (P > 0.05). Data are shown in Table 1.

Pathological analysis demonstrated a tiny inflammatory infiltration and small areas of bone loss close to the root apex on day 7. Mild inflammatory infiltration and large areas of bone loss were observed on day 14, which then increased quickly from day 21 to day 35. The extent of bone loss also enlarged rapidly with many continuous bone lacunae along the lesion edge. The lesion expansion stabilized with less absorbed lacunae from day 42 to day 49 (Figure 1(a)).

### 3.2. CypA, CD147, and MMP-9 Expression Dynamics in Mice Periapical Lesion

**Figure 2:**
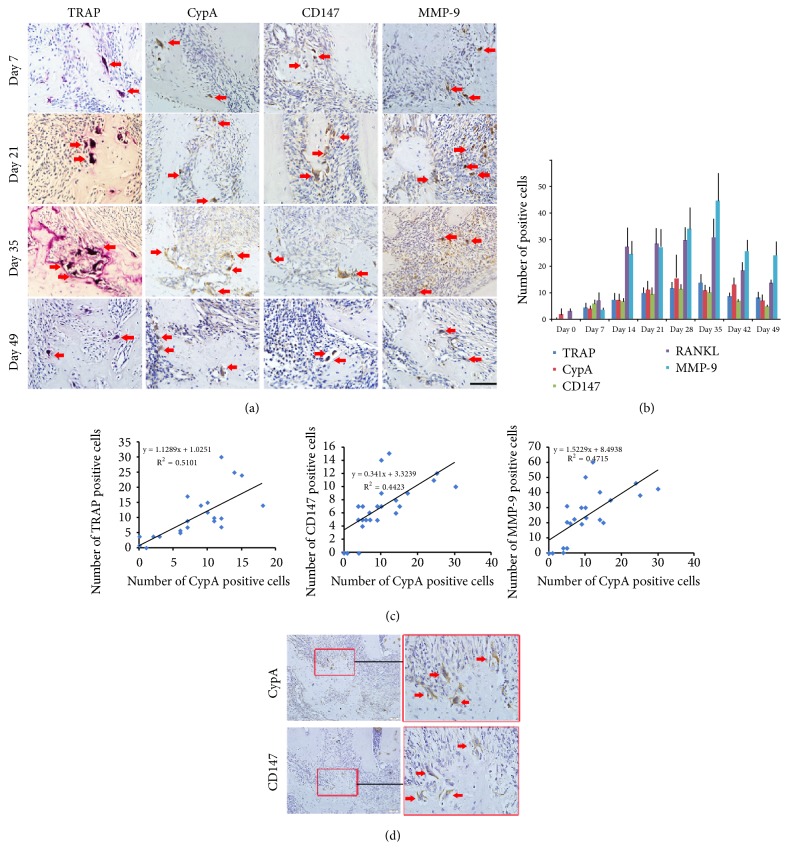
(a) The immunohistochemistry staining of TRAP, CypA, CD147, and MMP-9 on day 7, day 21, day 35, and day 49 in mice periapical lesions (original magnification, 400; scale bar = 50 *μ*m). The dark brown cells represented the positive cells. Along with the exacerbation of the inflammatory reaction in periapical lesion, the positive cells of TRAP, CypA, CD147, and MMP-9 increased until on day 35 and all decreased on day 49. The distribution of TRAP, CypA, and CD147 positive cells was mostly in osteoclast-like cells adjacent to the bone resorption lacuna (red arrows). (b) The number of TRAP, CypA, CD147, and MMP-9 positive cells in periapical lesions. (c) The correlation between CypA^+^ and TRAP^+^, MMP-9^+^, and CD147^+^ cells in mice periapical lesions, respectively. (d) The colocalization of CypA and CD147 in seriate sections of periapical lesions. The majority of the positive cells for CypA/CD147 were multinucleated osteoclast-like cells showed by red arrows.

On day 0, no CypA+ cells or CD147+ cells could be clearly detected. Some CypA+ cells appeared on day 7, increased rapidly until day 28, and decreased until day 49, similar to that of CD147+ cells (Figures 2(a) and 2(b)). CypA mainly expressed in multinucleated osteoclast-like cells, lymphocytes, osteoblast-like cells, macrophages, and fibroblasts; and CD147 mainly expressed in multinucleated osteoclast-like cells, osteoblast-like cells, macrophages, and fibroblasts. Significant correlation was observed between CypA+ and CD147+ cells from day 0 to day 49 (*R*^2^ = 0.4423, P < 0.01, Figure 2(c)). Expression of MMP-9 exhibited similar trend to that of CypA/CD147 signaling. On day 0, no MMP-9-positive cells could be obviously detected. The number of MMP-9-positive cells remarkably increased from day 7 to day 14, peaked at day 35, and decreased until day 49 (Figures 2(a) and 2(b)). Significant correlation was observed from day 0 to day 49 between CypA-positive cells and MMP-9-positive cells (*R*^*2*^ = 0.4715, P < .01, Figure 2(c)). Data are shown in Table 1.

TRAP staining showed that few osteoclasts could be seen in the apical region on day 7. The number of osteoclasts increased from day 7 to day 21, peaked on day 35, and then showed a continuous rate of decrease from day 35 to day 49 (Figure 2(a)). And there was a significant positive correlation between CypA-positive cells and TRAP-positive osteoclasts (*R*^2^ = 0.5101, P < 0.01, Figure 2(c)). Additionally, Serial sections confirmed that the colocalization of CypA and CD147 was mostly in multinucleated osteoclast-like cells, macrophages, and fibroblasts adjacent to the bone with immunohistochemical staining (Figure 2(d)). Data are shown in Table 1.

### 3.3. Immunofluorescent Colocalization of CypA and MMP-9 in Mouse Periapical Lesion

**Figure 3:**
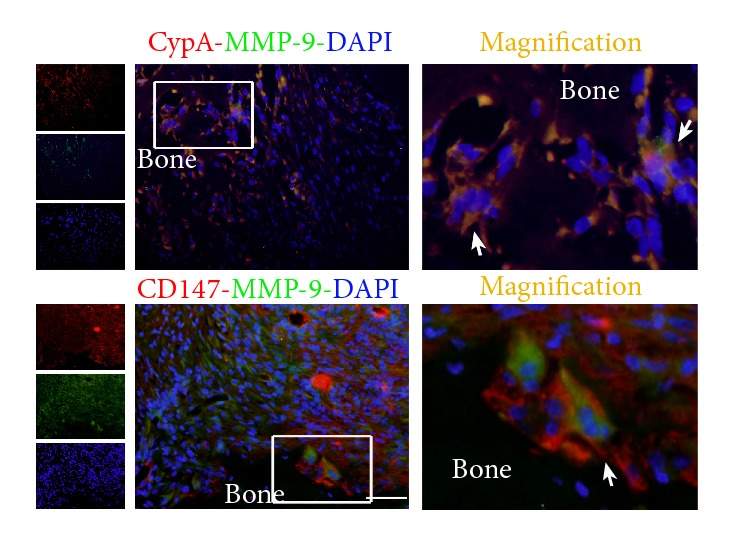
Double immunofluorescence staining of CypA or CD147 with MMP-9. MMP-9 was stained with Dylight488 (green), and CypA or CD147 was stained with CY3 (red). Nuclei were counterstained with DAPI (blue). The distribution of CypA-positive or CD147-positive cells was overlapped with MMP-9-positive cells. The double staining cells were mainly osteoclast, macrophages, and fibroblasts. The arrows indicated the colocalization of CypA or CD147 with MMP-9 on osteoclasts-like cells on the bone surface. Magnification: 400×; Scale bar = 50 *μ*m.

Colocalization of CypA or CD147 with MMP-9 was performed with double immunofluorescence staining in Serial sections on days 35. The immunofluorescence analysis showed that osteoclast, macrophages, and fibroblasts were CypA/CD147 expressing cells in the progress of periapical lesions. MMP-9-positive cells were widely distributed in periapical lesions. A number of CypA-positive or CD147-positive cells were overlapped with MMP-9-positive cells; and a number of CypA-positive and CD147-positive cells were both located near the bone surface. The double staining cells were mainly osteoclasts-like and shuttle type cells adjacent to the bone surface (Figure 3).

### 3.4. The Protein Expression of CD147 and MMP-9 in CypA Inhibitor-Treated RAW264.7 Cells

**Figure 4:**
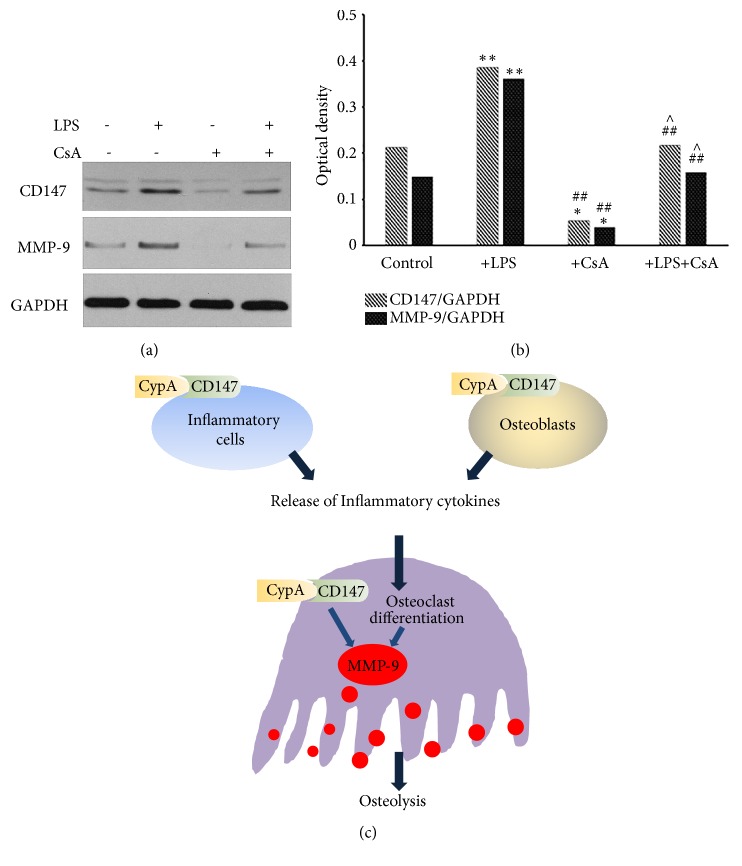
The protein expression of CD147 and MMP-9 in CypA inhibitor-treated RAW 264.7 cells. (a) Western bolt analysis showed the different expression of CD147 and MMP-9 under different material treatment. The protein expression of CD147 and MMP-9 were both down-regulated by CsA treatment in RAW 264.7 cells. (b) The ratio of CD147/GADPH and MMP-9/GADPH in different group was showed by bar chart. *∗*P < 0.05 versus control group; ^#^P < 0.05 versus LPS-treated group; ^∧^P < 0.05 versus CsA-treated group. (c) The role of CypA and CD147 in periapical bone destruction. Released CypA interacts with CD147, which can be found on the surface of inflammatory cells, osteoclasts, and osteoblasts, accelerating inflammatory cytokines production and bone resorption.

Under LPS stimulation, the protein expression of CD147 and MMP-9 in RAW 264.7 cells was both upregulated compared with control group (P < 0.05). However, after the intervention of CypA inhibitor, CD147 and MMP-9 expressions were obviously downregulated compared with LPS stimulation group (P < 0.05) (Figures 4(a) and 4(b)).

## 4. Discussion

CypA/CD147 complex is a regulator of inflammatory responses which strongly induces the migration of monocytes/macrophages and promotes the secretion of MMP-9, IL-6, and TNF-*α* [28]. Recent studies confirmed that CypA/CD147 complex functions in inflammatory diseases, including periodontitis, RA, and atherosclerosis [10, 20, 23]. Therefore, we hypothesized that CypA/CD147 may play a role in the pathogenesis of periapical tissues. And a mouse periapical lesion model was employed by pulp exposure to investigate the specific expression of CypA and CD147. Our study selected a long study period starting from day 0 to day 49 to observe the periapical lesion development, thereby providing indispensable additional information to mice periapical lesion study.

CypA, a widely distributed intracellular protein, is secreted by various cells in response to inflammatory stimuli [29]. Our immunohistochemical experiment results demonstrated that CypA was overexpressed in the development of periapical lesions. Interestingly, multinucleated osteoclast-like cells presented strong CypA positivity on the surface of the eroded alveolar bone surface. Correlation analysis revealed the positive relationship between CypA+ cells and TRAP+ cells. Previous studies have also reported that CypA expression localizes in osteoclasts and participates in the alveolar bone destruction in periodontitis [23]. However, one study showed that CypA dually exhibits proosteogenic and antiosteoclastic functions with CypA-knockout mice [15], which is contrary to the present result. The difference is possibly due to the pathological environment; the present investigation focused on the role of CypA in inflammation-mediated disease but not on noninflammatory physiological condition. Macrophages and fibroblasts also expressed CypA in mice periapical lesions. Coincident with previous studies in cardiovascular diseases and rheumatoid arthritis, multiple inflammatory cells such as monocytes, macrophages, fibroblasts, and endothelial cells assembled and secreted CypA during diseases development [10, 13, 17, 30]. On the basis of previous findings and our research, the participation of CypA in the pathogenesis of periapical lesion may be one of the factors responsible for osteoclasts activation and inflammatory cells assembling and infiltration.

As an extracellular matrix metalloproteinase inducer, CD147 is thought to be the main cell surface receptor mediating CypA signal transduction [28]. CypA overexpression was observed to increase CD147 and MMP-9 expression in a concentration and time-dependent manner [24]. Consistent with previous study, CD147 was found in high levels in inflamed periapical lesion and also mainly overexpressed in multinucleated osteoclasts similar to CypA in present study [5]. Serial sections further confirmed the colocalization of CypA and CD147 on osteoclast with immunohistochemical staining. CypA/CD147 signaling was reported to be responsible for the destruction of cartilage and bone in patients with RA, intervention of which evidently reduced the RA development [10, 28]. In the present study, inflammatory cells including macrophages and fibroblasts were CypA/CD147 signaling expressing cells, and the dynamic expression of CypA and CD147 was consistent with osteoclast number and periapical bone resorption area, indicating the important roles of CypA/CD147 signaling in inflammatory cells infiltration and osteoclastogenesis during periapical lesions progression.

Mature osteoclasts secrete hydrogen ions and proteinases such as MMP-9 to achieve extracellular matrix and bone degradation [31, 32]. Inhibition of MMP-9 could downregulate the expression of osteoclast maturation genes and suppress the osteoclastic pit formation [31, 33]. Moreover, MMP-9 expression was reported to be upregulated via CypA/CD147 signaling pathway and involve in the bone destruction of RA patients [17]. When CypA/CD147 signaling was blocked, MMP-9 expression was downregulated accordingly [21]. In the present study, high MMP-9 expression levels were observed and also exhibited colocalization with CypA and CD147 expression mainly in the multinucleated osteoclasts, macrophages, and fibroblasts of the periapical lesions. And under CypA inhibitor (CsA, an inhibitor of CypA has been commonly and widely used in a lot of fields, such as virus infection, inflammation, metabolism, and cancer [9, 34–36]) treatment, the protein expression of CD147 and MMP-9 both decreased in LPS-stimulated RAW264.7 cells. Previous studies have reported that CypA acted as an important mediator in accelerating MMP-9 and CD147 expression. Combined with the results above, we speculated that CypA/CD147 signaling activation probably played a key role in promoting osteoclastogenesis, inflammatory cells infiltration, and MMP-9 production, thus leading to bone resorption during periapical lesion progression. Meanwhile, we would conduct further research of using specific CypA inhibitor to confirm the role of CypA in the regulation of osteoclasts activation in vitro.

Collectively, our findings showed for the first time that CypA/CD147 signaling was activated and strongly associated with osteoclast formation and MMP-9 expression during the pathogenesis of periapical lesions. Therefore, intervention of CypA/CD147 signaling could probably provide a potential therapeutic target for attenuating inflammatory periapical bone resorption. Further investigation in contributing to the pathogenesis of periapical lesions induced by CypA/CD147 signaling needs to be carried on in the future.

## Figures and Tables

**Table 1:** Numbers of CypA^+^ Cells, CD147^+^ cells, and MMP-9^+^cells per high-power field (*∗* 400) and percentage of osteoclasts, bone resorption volumes, and surface of periapical lesions (mean ± standard deviation).

GROUP	N	CypA^+^ cells/hpf	CD147^+^ cells/hpf^*ᴥ*^	TRAP^+^ cells/hpf^*ԓ*^	MMP-9^+^ cells/hpf^*⨲*^	Lesion Volume (mm^3^)	Lesion Surface (mm^2^)^ ф^
Day 0	5	1.67 ± 2.08^§ф*ɣǂ*^	----^*‖*§ф*ɣǂ*†^	0.14 ± 0.38^§ф*ɣǂϮ*^	----^*‖*§ф*ɣǂϮ*^	0.063 ± 0.012^ф*ɣǂϮ*^	0.022 ± 0.011^ф*ɣǂϮ*^
Day 7	5	3.75 ± 1.26^§ф*ɣǂ*^	5.67 ± 1.54	4.17 ± 1.72^§ф*ɣǂ*^	3.67 ± 0.58^*‖*§ф*ɣǂϮ*^	0.088 ± 0.038^*ɣǂϮ*^	0.023 ± 0.009^ф*ɣǂϮ*^
Day 14	5	7.17 ± 2.31^§ф*ɣǂ*^	6.5 ± 1.29^†^	7.22 ± 2.60^ф*ɣ*^	24.6 ± 4.83^‡†ф*ɣ*^	0.133 ± 0.045^*ɣǂϮ*^	0.031 ± 0.007^*ɣǂϮ*^
Day 21	5	11.16 ± 3.25^†‡ф*‖*^	9.33 ± 2.58^†^	9.88 ± 2.03^†‡^	27.25 ± 6.85^‡†*ɣ*^	0.157 ± 0.067^*ɣǂϮ*^	0.035 ± 0.006^*ɣǂϮ*^
Day 28	5	15.56 ± 8.59^†‡*‖*§*ɣǂ*^	11.25 ± 1.83^†*Ϯ*^	11.71 ± 2.21^†‡^	34.13 ± 8.22^‡†*‖ǂϮ*^	0.257 ± 0.064^†^	0.048 ± 0.013^†‡^
Day 35	5	10.78 ± 2.22^†‡*‖*ф^	10.1 ± 2.30^†*Ϯ*^	13.8 ± 3.03^†‡^	44.67 ± 10.28^*‖*§*ǂϮ*†‡^	0.316 ± 0.086^†‡*‖*§^	0.049 ± 0.008^†‡*‖*§^
Day 42	5	13 ± 2.65^†‡*‖*ф^	6.67 ± 0.58^†^	8.67 ± 1.63^†‡^	25.57 ± 4.12^‡*ɣ*†ф^	0.351 ± 0.043^†‡*‖*§^	0.055 ± 0.005^†‡*‖*§^
Day 49	5	7 ± 2^†ф*ǂ*^	4.67 ± 0.58^†ф*Ϯ*^	8 ± 2.35^†^	24 ± 5.29^‡*ɣ*†ф^	0.349 ± 0.014^†‡*‖*§^	0.058 ± 0.004^†‡*‖*§^

The greatest numbers of CypA +cells and CD147+ cells per high-power field appeared on day 28. The greatest percentage of MMP-9+ cells and osteoclasts reached on day 35. The greatest lesion volume appeared on day 42 and the greatest lesion surface appeared on day 49. Significant differences were found between day 0 and days 21, 28, 35, 42, and 49 and between day 7 and day 28 values of CypA+ cells. Statistical significance between groups was showed by tags. Positive correlation was found between lesion volume and surface and between numbers of CypA+ cells, CD147+ cells, MMP-9+ cells, and osteoclasts. hpf, high-power field.

^*ԓ*^Positive correlation (R^2^ = 0.5101, P < .01) with number of CypA + cells/hpf.

^*ᴥ*^Positive correlation (R^2^ =0.4423, P < .01) with number of CypA + cells/hpf.

^*⨲*^ Positive correlation (R^2^ = 0.4715, P < .01) with number of CypA + cells/hpf.

^ф^Positive correlation (R^2^ = 0.4053, P < .01) with lesion volume.

^‡^P < .05 versus 7-day group.

^*‖*^P < .05 versus 14-day group.

^§^P < .05 versus 21-day group.

^ф^P < .05 versus 28-day group.

^*ɣ*^P < .05 versus 35-day group.

^*ǂ*^P < .05 versus 42-day group.

^*Ϯ*^P < .05 versus 49-day group.

^†^P < .05 versus 0-day group (control).

## Data Availability

(1) The data [Table 1] used to support the findings of this study are included within the article. (2) Previously reported data [Materials and Methods] were used to support this study and are available at DOI: 10.1016/j.joen.2014.10.010, DOI: 10.3390/nu10040472, and DOI: 10.1111/iej.12258. These prior studies (and datasets) are cited at relevant places within the text as [24–26]. (3) The data [original date] used to support the findings of this study are available from the corresponding author upon request.
